# Supplementary material for: Role and mechanism of NCAPD3 in promoting malignant behaviors in gastric cancer
Source: Front Pharmacol. 2024 Apr 22;15:1341039. doi: 10.3389/fphar.2024.1341039 (PMC11070777; doi:10.3389/fphar.2024.1341039)
Supplement: Supplementary file 11 [file DataSheet2.ZIP › GSEA/Transcipt factor/my_analysis.Gsea.1599461814734/HES2_TARGET_GENES.html]

Details for gene set HES2\_TARGET\_GENES[GSEA]

|  || Dataset | filtered\_dataset.sample\_info.cls#WT\_versus\_NCAPD3\_MUT |
| Phenotype | sample\_info.cls#WT\_versus\_NCAPD3\_MUT |
| Upregulated in class | NCAPD3\_MUT |
| GeneSet | HES2\_TARGET\_GENES |
| Enrichment Score (ES) | -0.2421317 |
| Normalized Enrichment Score (NES) | -1.9903883 |
| Nominal p-value | 0.0 |
| FDR q-value | 0.06710431 |
| FWER p-Value | 0.611 |
Table: GSEA Results Summary

  

Fig 1: Enrichment plot: HES2\_TARGET\_GENES      
 Profile of the Running ES Score & Positions of GeneSet Members on the Rank Ordered List

  

| SYMBOL | TITLE | RANK IN GENE LIST | RANK METRIC SCORE | RUNNING ES | CORE ENRICHMENT || 1 | 63926 | ANKEF1 | 4 | 1.180 | 0.0130 | No |
| 2 | 2072 | ERCC4 | 7 | 1.130 | 0.0269 | No |
| 3 | 342184 | FMN1 | 9 | 1.110 | 0.0413 | No |
| 4 | 2243 | FGA | 18 | 1.039 | 0.0493 | No |
| 5 | 7763 | ZFAND5 | 26 | 1.006 | 0.0576 | No |
| 6 | 51279 | C1RL | 31 | 0.991 | 0.0680 | No |
| 7 | 132320 | SCLT1 | 56 | 0.902 | 0.0617 | No |
| 8 | 2120 | ETV6 | 63 | 0.862 | 0.0688 | No |
| 9 | 4204 | MECP2 | 70 | 0.856 | 0.0758 | No |
| 10 | 56288 | PARD3 | 99 | 0.790 | 0.0648 | No |
| 11 | 55687 | TRMU | 128 | 0.774 | 0.0536 | No |
| 12 | 6925 | TCF4 | 132 | 0.768 | 0.0618 | No |
| 13 | 83877 | TM2D2 | 173 | 0.704 | 0.0402 | No |
| 14 | 613 | BCR | 180 | 0.695 | 0.0451 | No |
| 15 | 6672 | SP100 | 185 | 0.689 | 0.0514 | No |
| 16 | 400410 | ST20 | 235 | 0.647 | 0.0221 | No |
| 17 | 9778 | KIAA0232 | 247 | 0.633 | 0.0222 | No |
| 18 | 2632 | GBE1 | 280 | 0.609 | 0.0056 | No |
| 19 | 54758 | KLHDC4 | 292 | 0.598 | 0.0052 | No |
| 20 | 129831 | RBM45 | 310 | 0.587 | -0.0000 | No |
| 21 | 4297 | KMT2A | 337 | 0.573 | -0.0125 | No |
| 22 | 8573 | CASK | 368 | 0.554 | -0.0282 | No |
| 23 | 1183 | CLCN4 | 373 | 0.551 | -0.0238 | No |
| 24 | 90102 | PHLDB2 | 384 | 0.546 | -0.0241 | No |
| 25 | 104355135 | LINC00431 | 401 | 0.535 | -0.0293 | No |
| 26 | 84274 | COQ5 | 419 | 0.523 | -0.0354 | No |
| 27 | 9075 | CLDN2 | 420 | 0.522 | -0.0282 | No |
| 28 | 805 | CALM2 | 423 | 0.518 | -0.0227 | No |
| 29 | 85015 | USP45 | 434 | 0.513 | -0.0235 | No |
| 30 | 6256 | RXRA | 439 | 0.509 | -0.0196 | No |
| 31 | 8607 | RUVBL1 | 451 | 0.506 | -0.0213 | No |
| 32 | 2920 | CXCL2 | 454 | 0.501 | -0.0160 | No |
| 33 | 4191 | MDH2 | 494 | 0.476 | -0.0399 | No |
| 34 | 4091 | SMAD6 | 519 | 0.462 | -0.0522 | No |
| 35 | 55245 | UQCC1 | 580 | 0.434 | -0.0930 | No |
| 36 | 55181 | SMG8 | 602 | 0.422 | -0.1036 | No |
| 37 | 9842 | PLEKHM1 | 614 | 0.416 | -0.1065 | No |
| 38 | 10061 | ABCF2 | 626 | 0.408 | -0.1095 | No |
| 39 | 286410 | ATP11C | 637 | 0.405 | -0.1117 | No |
| 40 | 55750 | AGK | 697 | 0.380 | -0.1525 | No |
| 41 | 84159 | ARID5B | 699 | 0.379 | -0.1481 | No |
| 42 | 23741 | EID1 | 741 | 0.355 | -0.1751 | No |
| 43 | 64087 | MCCC2 | 747 | 0.352 | -0.1742 | No |
| 44 | 404636 | FAM45A | 764 | 0.341 | -0.1820 | No |
| 45 | 10492 | SYNCRIP | 779 | 0.334 | -0.1883 | No |
| 46 | 1973 | EIF4A1 | 804 | 0.319 | -0.2027 | No |
| 47 | 7037 | TFRC | 824 | 0.303 | -0.2133 | No |
| 48 | 4071 | TM4SF1 | 851 | -0.251 | -0.2301 | No |
| 49 | 360 | AQP3 | 857 | -0.263 | -0.2304 | No |
| 50 | 2872 | MKNK2 | 860 | -0.269 | -0.2283 | No |
| 51 | 4502 | MT2A | 863 | -0.277 | -0.2261 | No |
| 52 | 144195 | SLC2A14 | 872 | -0.283 | -0.2285 | No |
| 53 | 7296 | TXNRD1 | 877 | -0.289 | -0.2276 | No |
| 54 | 6520 | SLC3A2 | 878 | -0.290 | -0.2237 | No |
| 55 | 10589 | DRAP1 | 882 | -0.300 | -0.2219 | No |
| 56 | 4008 | LMO7 | 909 | -0.335 | -0.2375 | Yes |
| 57 | 3164 | NR4A1 | 912 | -0.341 | -0.2344 | Yes |
| 58 | 301 | ANXA1 | 914 | -0.342 | -0.2305 | Yes |
| 59 | 4052 | LTBP1 | 928 | -0.353 | -0.2358 | Yes |
| 60 | 2317 | FLNB | 936 | -0.360 | -0.2364 | Yes |
| 61 | 153830 | RNF145 | 940 | -0.363 | -0.2337 | Yes |
| 62 | 8780 | RIOK3 | 948 | -0.367 | -0.2342 | Yes |
| 63 | 79094 | CHAC1 | 949 | -0.367 | -0.2292 | Yes |
| 64 | 9052 | GPRC5A | 951 | -0.369 | -0.2249 | Yes |
| 65 | 10241 | CALCOCO2 | 954 | -0.373 | -0.2213 | Yes |
| 66 | 83641 | FAM107B | 960 | -0.381 | -0.2200 | Yes |
| 67 | 7067 | THRA | 976 | -0.392 | -0.2264 | Yes |
| 68 | 84919 | PPP1R15B | 979 | -0.394 | -0.2225 | Yes |
| 69 | 9180 | OSMR | 981 | -0.394 | -0.2179 | Yes |
| 70 | 5163 | PDK1 | 982 | -0.394 | -0.2125 | Yes |
| 71 | 25902 | MTHFD1L | 986 | -0.395 | -0.2095 | Yes |
| 72 | 3176 | HNMT | 991 | -0.398 | -0.2071 | Yes |
| 73 | 901 | CCNG2 | 998 | -0.405 | -0.2063 | Yes |
| 74 | 6286 | S100P | 1011 | -0.412 | -0.2100 | Yes |
| 75 | 10938 | EHD1 | 1019 | -0.419 | -0.2097 | Yes |
| 76 | 966 | CD59 | 1040 | -0.436 | -0.2193 | Yes |
| 77 | 54676 | GTPBP2 | 1050 | -0.447 | -0.2202 | Yes |
| 78 | 7132 | TNFRSF1A | 1055 | -0.449 | -0.2172 | Yes |
| 79 | 6590 | SLPI | 1062 | -0.453 | -0.2157 | Yes |
| 80 | 8609 | KLF7 | 1071 | -0.457 | -0.2156 | Yes |
| 81 | 1604 | CD55 | 1090 | -0.468 | -0.2233 | Yes |
| 82 | 3669 | ISG20 | 1097 | -0.475 | -0.2214 | Yes |
| 83 | 857 | CAV1 | 1098 | -0.475 | -0.2149 | Yes |
| 84 | 3556 | IL1RAP | 1099 | -0.476 | -0.2084 | Yes |
| 85 | 8864 | PER2 | 1109 | -0.483 | -0.2088 | Yes |
| 86 | 7378 | UPP1 | 1111 | -0.484 | -0.2030 | Yes |
| 87 | 2729 | GCLC | 1123 | -0.497 | -0.2048 | Yes |
| 88 | 55370 | PPP4R1L | 1124 | -0.497 | -0.1980 | Yes |
| 89 | 54541 | DDIT4 | 1133 | -0.502 | -0.1973 | Yes |
| 90 | 604 | BCL6 | 1136 | -0.504 | -0.1920 | Yes |
| 91 | 55124 | PIWIL2 | 1154 | -0.516 | -0.1982 | Yes |
| 92 | 54407 | SLC38A2 | 1182 | -0.553 | -0.2116 | Yes |
| 93 | 8535 | CBX4 | 1184 | -0.555 | -0.2048 | Yes |
| 94 | 51776 | ZAK | 1196 | -0.571 | -0.2056 | Yes |
| 95 | 23657 | SLC7A11 | 1205 | -0.581 | -0.2039 | Yes |
| 96 | 23461 | ABCA5 | 1206 | -0.583 | -0.1959 | Yes |
| 97 | 4651 | MYO10 | 1213 | -0.588 | -0.1925 | Yes |
| 98 | 29968 | PSAT1 | 1215 | -0.589 | -0.1852 | Yes |
| 99 | 10221 | TRIB1 | 1229 | -0.604 | -0.1871 | Yes |
| 100 | 4301 | MLLT4 | 1237 | -0.614 | -0.1841 | Yes |
| 101 | 26137 | ZBTB20 | 1244 | -0.622 | -0.1803 | Yes |
| 102 | 837 | CASP4 | 1245 | -0.623 | -0.1718 | Yes |
| 103 | 5187 | PER1 | 1254 | -0.641 | -0.1693 | Yes |
| 104 | 2355 | FOSL2 | 1262 | -0.648 | -0.1658 | Yes |
| 105 | 55638 | SYBU | 1275 | -0.663 | -0.1661 | Yes |
| 106 | 7994 | KAT6A | 1279 | -0.666 | -0.1593 | Yes |
| 107 | 83931 | STK40 | 1281 | -0.669 | -0.1510 | Yes |
| 108 | 54800 | KLHL24 | 1287 | -0.677 | -0.1456 | Yes |
| 109 | 202 | AIM1 | 1290 | -0.681 | -0.1378 | Yes |
| 110 | 57761 | TRIB3 | 1292 | -0.685 | -0.1293 | Yes |
| 111 | 3084 | NRG1 | 1320 | -0.739 | -0.1402 | Yes |
| 112 | 10349 | ABCA10 | 1326 | -0.747 | -0.1339 | Yes |
| 113 | 84803 | GPAT3 | 1327 | -0.748 | -0.1236 | Yes |
| 114 | 10611 | PDLIM5 | 1335 | -0.758 | -0.1187 | Yes |
| 115 | 1649 | DDIT3 | 1341 | -0.779 | -0.1119 | Yes |
| 116 | 80709 | AKNA | 1351 | -0.795 | -0.1081 | Yes |
| 117 | 115123 | MARCH3 | 1360 | -0.828 | -0.1030 | Yes |
| 118 | 54790 | TET2 | 1364 | -0.842 | -0.0938 | Yes |
| 119 | 4734 | NEDD4 | 1369 | -0.856 | -0.0852 | Yes |
| 120 | 388743 | CAPN8 | 1375 | -0.899 | -0.0768 | Yes |
| 121 | 26298 | EHF | 1378 | -0.919 | -0.0658 | Yes |
| 122 | 10397 | NDRG1 | 1380 | -0.925 | -0.0540 | Yes |
| 123 | 23650 | TRIM29 | 1384 | -0.951 | -0.0433 | Yes |
| 124 | 59277 | NTN4 | 1390 | -0.999 | -0.0335 | Yes |
| 125 | 26959 | HBP1 | 1395 | -1.073 | -0.0219 | Yes |
| 126 | 1475 | CSTA | 1400 | -1.162 | -0.0092 | Yes |
| 127 | 3329 | HSPD1 | 1402 | -1.183 | 0.0062 | Yes |
Table: GSEA details [plain text format]

  

Fig 2: HES2\_TARGET\_GENES      
 Blue-Pink O' Gram in the Space of the Analyzed GeneSet

  

Fig 3: HES2\_TARGET\_GENES: Random ES distribution      
 Gene set null distribution of ES for **HES2\_TARGET\_GENES**
